# Joint emulation of Earth System Model temperature-precipitation realizations with internal variability and space-time and cross-variable correlation: fldgen v2.0 software description

**DOI:** 10.1371/journal.pone.0223542

**Published:** 2019-10-04

**Authors:** Abigail Snyder, Robert Link, Kalyn Dorheim, Ben Kravitz, Ben Bond-Lamberty, Corinne Hartin

**Affiliations:** 1 Joint Global Change Research Institute, Pacific Northwest National Laboratory, College Park, MD, United States of America; 2 Department of Earth and Atmospheric Sciences, Indiana University, Bloomington, IN, United States of America; 3 Atmospheric Sciences and Global Change Division, Pacific Northwest National Laboratory, Richland, WA, United States of America; Texas A&M University, UNITED STATES

## Abstract

Earth System Models (ESMs) are excellent tools for quantifying many aspects of future climate dynamics but are too computationally expensive to produce large collections of scenarios for downstream users of ESM data. In particular, many researchers focused on the impacts of climate change require large collections of ESM runs to rigorously study the impacts to both human and natural systems of low-frequency high-importance events, such as multi-year droughts. Climate model emulators provide an effective mechanism for filling this gap, reproducing many aspects of ESMs rapidly but with lower precision. The fldgen v1.0 R package quickly generates thousands of realizations of gridded temperature fields by randomizing the residuals of pattern scaling temperature output from any single ESM, retaining the spatial and temporal variance and covariance structures of the input data at a low computational cost. The fldgen v2.0 R package described here extends this capability to produce joint realizations of multiple variables, with a focus on temperature and precipitation in an open source software package available for community use (https://github.com/jgcri/fldgen). This substantially improves the fldgen package by removing the requirement that the ESM variables be normally distributed, and will enable researchers to quickly generate covarying temperature and precipitation data that are synthetic but faithful to the characteristics of the original ESM.

## Introduction

Two important topics to researchers in earth sciences, future climate dynamics, and joint human-Earth system modeling are the effects of extreme events and uncertainty in climate impacts [1, 2]. Understanding the uncertainty around extreme events also has direct relevance for decision makers, the U.S. energy grid, and other stakeholders. Generally, high-resolution future climate scenarios can be used as input for different process-based or economic models to assess impacts of future climate on economic, land, energy, and other systems; however, thousands of such high-resolution future scenarios may be needed for a full and robust analysis of the impacts of low-frequency high-importance extreme events and associated uncertainties [3–5].

Process-rich Earth System Models (ESMs) are capable of providing the high-resolution future climate scenarios for impacts studies, but are too computationally expensive to directly produce hundreds or thousands of realizations needed to understand the impacts of extreme events. Climate model emulators attempt to solve this problem by approximating the output a climate model would have produced had it been run repeatedly for a specified scenario [6]. These emulators are computationally cheap to run, but typically capture only the mean response of the climate and little to none of the variability that would be present in a real ESM output. Attempts to add variability to such emulations often lose important spatial and temporal correlations that fundamentally define ESM patterns (such as the El Niño Southern Oscillation), or else cannot produce the number of realizations necessary for extreme event or uncertainty studies.

In fldgen v1.0, Link et al. [7] (Paper LS1 hereafter) have solved many of these problems for the case of global temperature. This method generates random realizations of temperature global gridded time series that retain the spatial and temporal variance and covariance structures of the input ESM data at a much lower computational cost compared to the ESMs being emulated. Crucially, fldgen v1.0 does so without placing *a priori* limits on the form of the correlation function and without using bootstrap resampling of existing ESM output [8–10].

Nevertheless, temperature is not the only important variable for quantifying the effects of future climate on human systems. Precipitation, for example, is of interest because of the serious impacts of extreme precipitation events, as the distribution of precipitation over space and time is a primary driver of extreme events like droughts and floods [11]. Moreover, the water cycle is a key component of research focused on water cycle impact on agriculture and water consumption in energy production [3, 5, 12].

Recent research has shown that modeling temperature and precipitation extremes independently mischaracterizes drought hazards as the covariance between climate variables is missed [13]. Therefore, an effective emulator of temperature and precipitation must jointly produce temperature and precipitation realizations that capture such covariance. Fldgen v2.0 is designed to add this capability, allowing researchers to jointly generate two-variable (e.g. joint temperature and precipitation) fields that not only retain the individual variables’ spatial and temporal variance and covariance structures, but also inter-variable covariance. This method is meant to be used on annual, global gridded temperature and precipitation data. While many interesting climatological and biological processes occur at daily or monthly scales, this annual timescale is often used by researchers in the impacts community focused on long-term effects. Many of these researchers also require global gridded realizations of ESM variables [3, 5, 14]. It is true that past modeling approaches such as the UKCP09 framework have provided a methodology for stochastically generating daily weather data for specific regions that does to some degree account for correlations between multiple variables and has accurate statistical structure to high order [15]. However, because the focus of that method is on weather in specific regions, it does not necessarily incorporate dynamics that govern larger spatiotemporal scales to be usable in a global gridded manner.

Fldgen v2.0 provides a tool for modelers to rigorously analyze the impacts of extreme events that previously could not be fully evaluated due to limited ESM realizations and region-to-region teleconnections that are not explicitly known globally, such as multi-year droughts across different regions. The objective of this paper is to describe the open-source and publicly available fldgen v2.0 R package work-flow, capabilities, and accessibility for community use (https://github.com/jgcri/fldgen) in the context of joint generation of temperature and precipitation fields. This includes an overview of the updates to the fldgen algorithm from version 1.0 to version 2.0. The theory that supports this extension should be applicable to arbitrary pairs of ESM variables, though this has not been tested and is not a central claim of this work.

## Extension from version 1.0 to 2.0 and architecture

The scientific details of generating new realizations of residuals from a training matrix of ESM residuals are detailed in the fldgen v1.0 model description Paper LS1. One key feature of the version 1.0 process is that the generated time series of temperature residuals in any given grid cell is approximately normally distributed. This is true irrespective of the distributions of input residuals, due to the way the algorithm sums up contributions from a large number of random components. As a result, the method will preserve the distribution of input residuals only if they are normally distributed.

**Fig 1:**
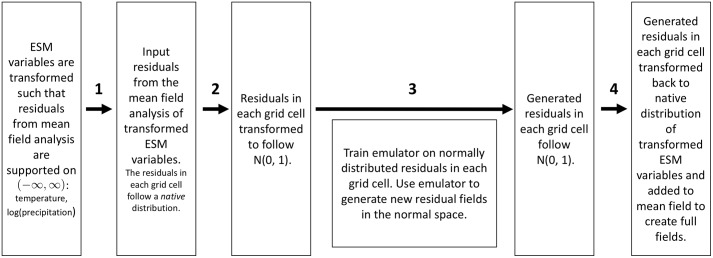
flgden v2.0 workflow. Workflow: Extending fldgen v1.0 (arrow 3) to fldgen v2.0 (arrows 1-4) for use with joint temperature and precipitation fields.

When the training data features normally distributed residuals in every grid cell, the field generating process generates new realizations of time series that preserves three key statistical properties of the training residuals (arrow 3 in Fig 1, Paper LS1):

**Statistical properties of training residuals preserved by version 1.0**
1. Distribution of values in a grid cell over time and between realizations. In other words, residuals in the grid cell are normally distributed with the same mean and variance as the training data in that grid cell.
2. Correlation between values in different grid cells.
3. Time autocorrelation of spatially correlated patterns of grid cells.

**Fig 2:**
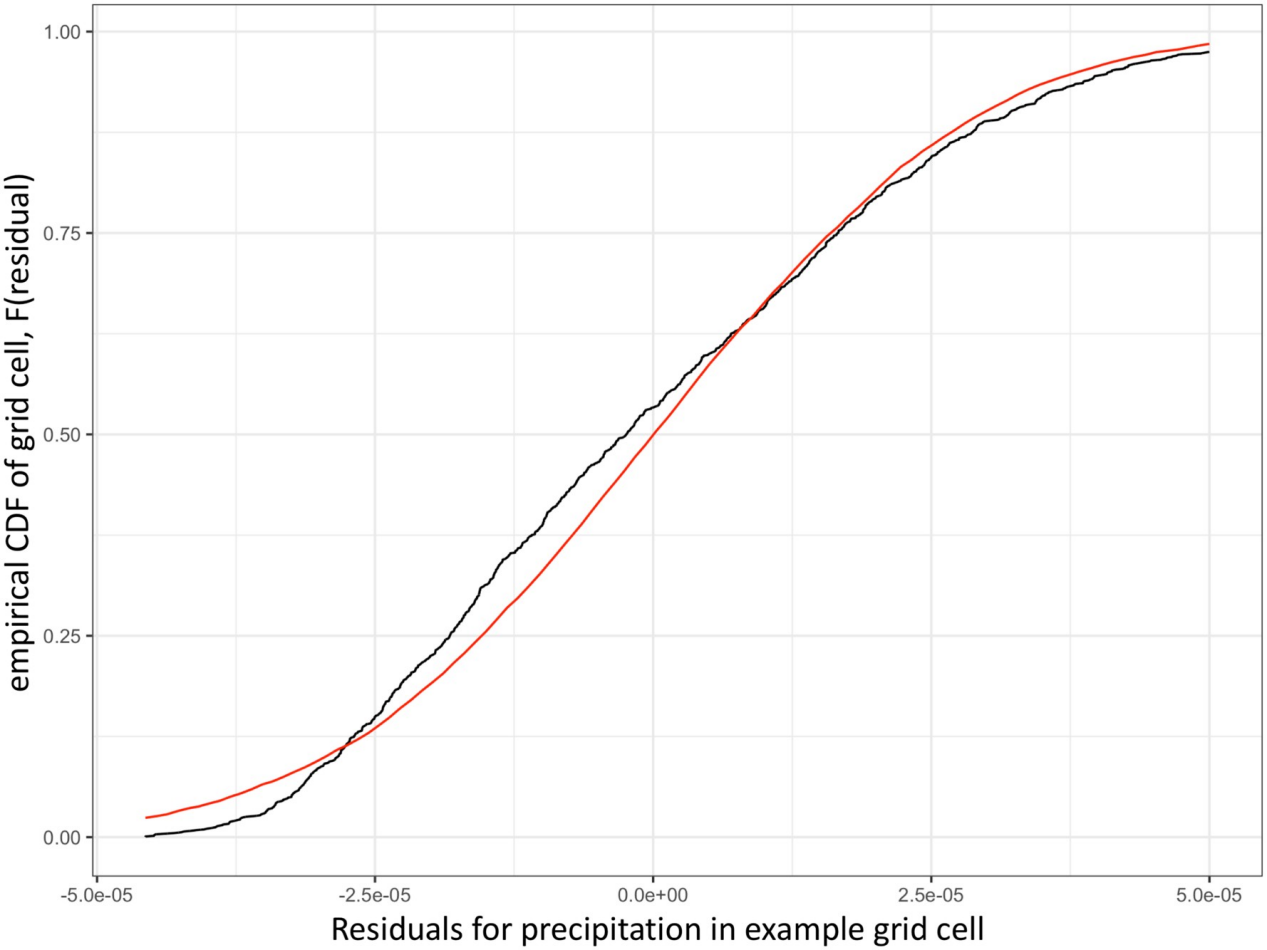
Comparison of empirical CDF from precipitation data with normal distribution. The empirical CDF of precipitation residuals in a single grid cell (black) and the CDF of a normal distribution with the same mean and variance as the precipitation residuals (red).

For many ESMs, the temperature residuals in each grid cell are indeed approximately normally distributed. However, residuals for other variables, such as precipitation, in many grid cells may have a non-normal distribution (e.g. Fig 2). Thus, in addition to the joint emulation of variables such as temperature and precipitation, one major goal of fldgen v2.0 is to make the field generation process more robust to non-normally distributed ESM variables. Even in cases where distributions are reasonably well approximated by normal distributions, this change removes the need for a user’s expert judgement as to whether an ESM variable’s residuals in each grid cell follow a distribution sufficiently close to normal to use the field generating method.

For the core field generating method, each ESM variable must effectively be able to accept residuals of between −∞ and ∞ for addition to its mean field. This is already the case for temperature and no transformation is needed. However, because precipitation values in an ESM cannot be negative, either the generated residuals have to be constrained to avoid negative precipitation while preserving the ESM spatiotemporal and intervariable statistics, or the method must operate on a transformation of precipitation that can accept residuals between −∞ and ∞. The latter is more straightforward, and so residuals are generated for log(precipitation) rather than precipitation. The transformation of generated full fields from log(precipitation) space back to precipitation space is trivial for log transformations. Indeed, any function that is continuous, invertible, strictly increasing, and results in a transformed ESM variable whose residuals are supported on (−∞, ∞) will preserve the ESM spatiotemporal statistical properties as desired.

Producing joint temperature and log(precipitation) residual realizations therefore requires the development of an algorithm to handle non-normality in transformed ESM training data. This algorithm extension for fldgen v2.0 involves additional transformations on the input to and output from the version 1.0 process (Table 1, Fig 1) in order to return generated realizations of joint temperature and log(precipitation) residuals to the native temperature and log(precipitation) distribution in each grid cell. One additional benefit is that this extension removes an assumption necessary to use version 1.0: we previously were restricted to assuming temperature residuals followed a normal distribution in each grid cell. With version 2.0, it no longer matters if this is the case for ESM variables under consideration.

**Table 1:** Summary of the fldgen v2.0 algorithm. Details of the steps shared with version 1.0 (denoted with *) are provided in Paper LS1 [7].

1.*	Select ESM runs for training the emulator.
2.*	Select and fit the mean response model relating local temperature (*T*) and log-precipitation (log(*P*)) to global average temperature.
3.*	Calculate the residuals by subtracting mean response from ESM output.
4. *	Map the distribution of (*T* or log(*P*)) residuals in each grid cell from the *native* distribution of the grid cell to the standard normal distribution (*N*(0, 1)) via quantiles.
5.*	Form a joint matrix of state residuals (spatially flattened, concatenated, normally distributed *T* and log(*P*) residuals in each grid cell at each time, denoted by *x*(*t*)).
6*.	Perform principal components analysis (PCA) on the joint *T*-log(*P*) residual field state: $x\left(t\right)=\sum_{i=1}^{N}\phi_{i}\left(t\right){\hat{x}}_{i}$, where the ${\hat{x}}_{i}$ are the principal components, and the *ϕ*_*i*_(*t*) are the projection coefficients. This step expresses the grid state as a linear combination of orthogonal basis vectors that diagonalize the covariance matrix of the system to capture spatial correlations.
7.*	Compute the discrete Fourier transform (DFT) [16] of the residual field’s projection coefficients onto the principal components: $\Phi_{i}=F(\phi_{i}\left(t\right))$.
8.*	Choose new phases of Φ_*i*_ randomly, uniformly on [0, 2*π*) to create new frequency space coefficients $\Phi_{i}^{⋆}$ with $|\Phi_{i}\left|=\right|\Phi_{i}^{⋆}|$. This preserves the time autocorrelation function of the ESM training data by the Wiener-Khinchin Theorem [16].
9.*	Compute the projection coefficients $\phi_{i}^{⋆}$ of the new residual field as the inverse DFT of $\Phi_{i}^{⋆}$.
10.*	Compute the generated, joint *T*-log(*P*) residual field as $x^{⋆}\left(t\right)=\sum_{i=1}^{N}\phi_{i}^{⋆}\left(t\right){\hat{x}}_{i}$.
11.	Map the generated (*T* or log(*P*)) residuals in each grid cell back to the native (*T* or log(*P*)) distribution of that grid cell.
12.*	Add to the respective *T* or log(*P*) mean field to create the generated full fields for *T* and log(*P*).
13.	Take the exponential of the generated log(*P*) full fields to produce generated full fields of precipitation.

* Denotes a step shared with fldgen v1.0 and detailed in Paper LS1. The only differences in shared steps between version 1.0 and 2.0 are that the same step is applied to log(*P*) in addition to *T* in version 2.0.

The fldgen process, for both version 1.0 and 2.0, operates on a state vector, **x**(**t**). At any given time *t*, the state of the system is defined as the temperature residuals in every grid cell (flattened to one dimensional indices from the traditional latitude-longitude two dimensions), concatenated with the precipitation residuals in every grid cell if applicable (flattened to the same one dimensional spatial indices). Therefore, once the issues of non-normality are accounted for in the extension to version 1.0, cross-variable relationships are automatically captured and joint emulation is achieved.

We have developed a continuous, invertible, and strictly increasing transformation method that allows us to map between the native distributions of residuals for each grid cell and a normal distribution of residuals for each grid cell. This transformation removes the need for user expertise as to whether residuals in every grid cell follow a distribution sufficiently close to a normal distribution for the field generating method outlined in Paper LS1 to work.

**Fig 3:**
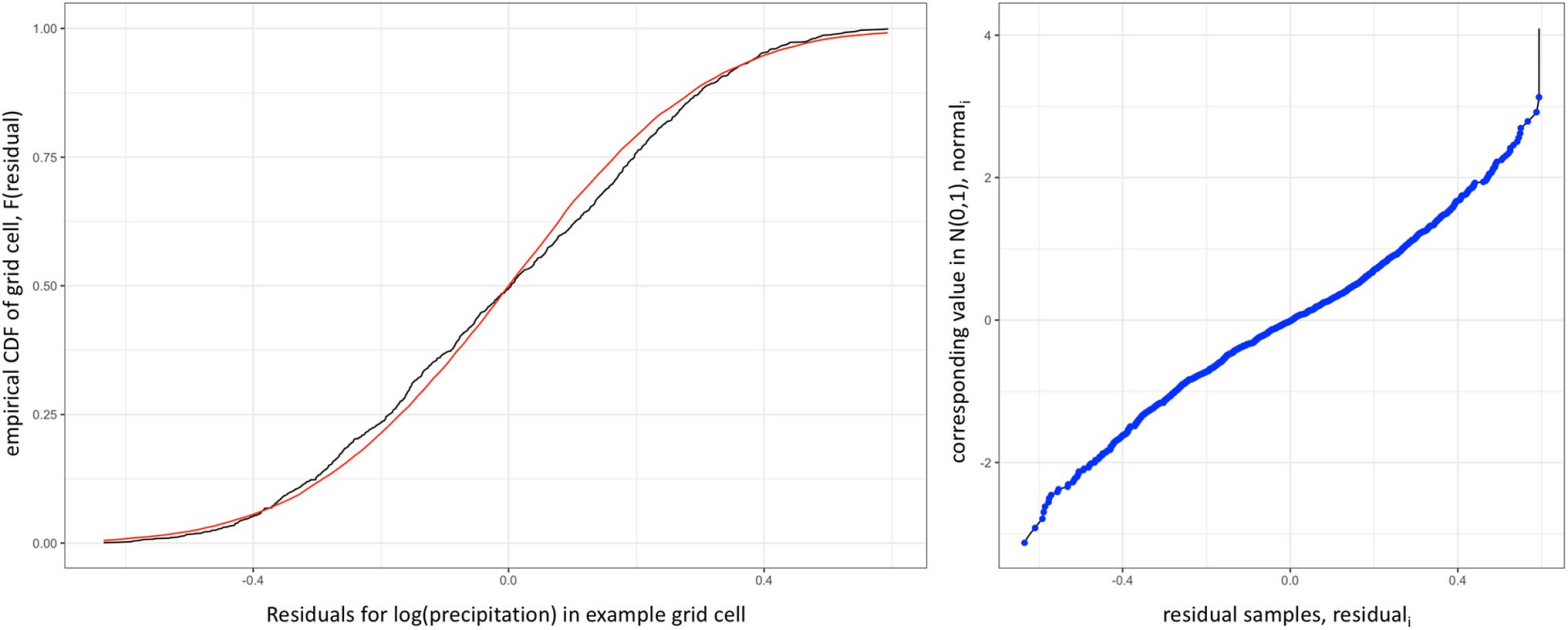
The constructed transformation from data’s native distribution to a normal distribution. Left: The empirical CDF of log(precipitation) residuals in the same grid cell as Fig 2 (black) and the CDF of a normal distribution with the same mean and variance as the log(precipitation) residuals (red). Right: The transformation between native and normally distributed residuals for this example grid cell. Note that this transformation is continuous, invertible, and strictly increasing.

For a given grid cell, the native distribution of residuals (temperature or precipitation) over time can be captured with an empirical cumulative distribution function (CDF), *F*(*x*) (Fig 3, left), which can then be used to return the quantile values corresponding to sampled residuals, *F*(*residual*_*i*_) = *quantile*_*i*_. These sampled quantile values are used to calculate the corresponding values sampled from the standard normal distribution, *N*(0, 1) (step 4 of the fldgen v2.0 algorithm summary in Table 1). Composing these steps leads to an increasing, continuous, invertible mapping between the native distribution of residuals in the grid cell and *N*(0, 1) (Fig 3, right), represented by arrow 2 of Fig 1.

Given a set of generated residuals that follow *N*(0, 1) for a single grid cell, mapping to the native distribution of residuals in the grid cell is a straightforward reversal of this process (captured in arrow 4 in Fig 1).

This mapping is applied to input, native residuals to create normally distributed residuals for emulator training (arrow 2 in Fig 1). The inverse of this mapping is applied to transform the generated, and therefore normally distributed, residuals for a grid cell back to the native, non-normal distribution initially sampled in **R** (arrow 4 in Fig 1). Further, because the mapping is continuous, invertible, and increasing, the rank-order correlation coefficient between residuals in two arbitrary grid cells is the same in the native distribution space as it is in the normal distribution. Combining this with the fact that the fldgen process generates new, normally distributed field of residuals (arrow 2 in Fig 1), we conclude that the rank-order correlation between input native values in different grid cells is the same as the rank-order correlation between between generated native values.

## Conclusions

Fldgen v2.0 provides a tool for climate change impacts modelers to rigorously analyze the impacts of extreme events that previously could not be fully evaluated due to limited ESM realizations and region-to-region teleconnections that are not explicitly known globally, such as multi-year droughts across different regions. With the mathematical construction described in the previous section and summarized in Fig 3 (right), when the full workflow depicted in Fig 1 is completed, the algorithm can be run on any input ESM residuals, provided their distribution in each grid cell is continuous and invertible. An emulator trained on these residuals can be used to generate new ESM residual time series that preserve all the same statistical properties preserved by version 1, with one small exception, viz. that in Property 2 the type of correlation preserved is the rank-order correlation, not necessarily the linear correlation coefficient. This change is a consequence of allowing arbitrary native distributions in the data. Additionally, the residual fields generated this way also preserve the inter-variable correlations present in the input data, a new property with no analog in version 1.

These generated residual time series are added to the mean fields for temperature and log(precipitation) to generate new full field time series. The transformation of the full field log(precipitation) time series values to precipitation values does not undo any of these statistical properties of the residual fields.

## Validation: Example data and automated testing

Extensive integrated, automatic testing is provided in the fldgen v2.0 R package to both check that the software performs as intended and to explicitly validate that the statistical properties of interest for included example ESM data (fldgen/inst/extdata) are indeed preserved in generated realizations. This explicit validation supplements mathematical proofs that the method being implemented in fldgen v2.0 has the described statistical preservation properties (further described and verified in Paper LS1).

The testing is automatically run when a Pull Request is opened in the fldgen GitHub repository and must be passed before the pull request can be merged. All checks were passed for the version of fldgen presented in this paper.

While it mathematically follows from the construction of this method that the rank-correlation coefficient is the same in generated data as it was in the training ESM data for every pairwise possibility of grid cell to grid cell comparison of temperature with temperature, precipitation with precipitation, and temperature with precipitation, we also performed an explicit check of of this using fldgen v2.0 emulator training and realization generating capabilities. This explicit test is not included in the automated testing suite as it takes many hours of computer time. An example trained emulator with raw ESM data, R scripts performing this test and the results are archived on zenodo (DOI 10.5281/zenodo.3372579) [17].

## Utility and limitations

The extension of fldgen from version 1.0 t0 version 2.0 has broadened the functionality from training using temperature data to joint training on temperature and precipitation by introducing a new algorithm for dealing with non-normality in the training data. Through this algorithm, any variable that scales with global mean temperature and whose residuals in each grid cell have a continuous, invertible, increasing empirical CDF could be jointly explored with temperature. Certainly this includes precipitation, but could easily be applied, as is, to variables such as relative humidity. Further, the version 1.0 requirement that input training data have approximately normally distributed residuals for the method to generate new residual fields with the correct spatiotemporal and cross-variable statistics has been removed. A user no longer has to judge whether their input training data is sufficiently close to normal to use fldgen.

Perhaps the most significant current limitation of fldgen is that its training data and thus outputs are limited to the annual time scale, while many interesting climatological and biological processes occur at daily or monthly scales—e.g. with respect to human health [18]. An expansion to sub-annual timescales is planned in future work, but appropriately capturing sub-annual ESM internal variability while accounting for the inherent seasonal signals in sub-annual ESM data for many variables is highly non-trivial.

A future extension to handle more than two variables jointly would also be straightforward by design. Thus there is wide potential for reuse of fldgen v2.0 for users of ESM outputs who require a large number of ESM datasets for research. The open-source nature of the software makes it particularly adaptable to a community user’s needs in a wide variety of ways.

## Installation

fldgen may be installed by using install_github from the devtools package.

## install the package if needed

devtools::install_github(‘JGCRI/fldgen’, ref = ‘v2.0.0 − rc.1’)

## Training the emulator from ESM data

As an open source R package, fldgen was implemented with a modular design so that different steps of the algorithm described in Table 1 can easily have different implementations. Using the R packages devtools and roxygen2, extensive documentation for every function created in the package is included. More detail of each step in Table 1 may be found in the tutorial vignettes included with the package (fldgen/vignettes/) and as described in Paper LS1. This subsection briefly demonstrates the function calls required for training an emulator and generating new realizations.

The user must select an ESM to emulate and provide annual, spatially disaggregated temperature and precipitation NetCDF files to be used for training. The fldgen package was designed with globally gridded data sets in mind, but would technically work with any spatial disaggregation. Currently, however, it is necessary that the training data be at an annual time scale. The training NetCDF files are most conveniently stored in some user-specified directory that can be passed directly to the fldgen training functions. Finally, all NetCDF files must follow CMIP5 naming conventions [19]. This is specified in the R manual and with informative error messages. Table 1 (right column), Steps 1-7 are performed by the function trainTP() in /R/trainTP.R, which is called as follows:

## load the package

library(‘fldgen’)

## specify the location of the training data

datadir <- ‘training/data/directory’

## train the emulator

trainTP(dat = datadir,

     tvarname = ‘tas’, tlatvar = ‘lat’, tlonvar = ‘lon’,

     tvarconvert_fcn = NULL,

     pvarname = ‘pr’, platvar = ‘lat’, plonvar = ‘on’,

     pvarconvert_fcn = log) ->

     emulator

Full details of the functions used internally by trainTP() are provided in the fldgen package vignettes.

### Description of inputs

The inputs to fldgen function trainTP are as follows:

dat A single directory name, or a list of NetCDF files. If a directory name is given, all NetCDF files in the directory will be used. The pairing of temperature and precipitation NetCDF files in the directory relies on the CMIP5 file naming conventions. Other naming conventions are not currently supported.
tvarname A string with the name of the temperature variable in the temperature NetCDF.
tlatvar A string with the name of the latitude coordinate variable in the temperature NetCDF files. Normally this is simply ‘lat’.
tlonvar A string with the name of the longitude coordinate variable in the temperature NetCDF files. Normally this is simply ‘lon’.
tvarconvert_fcn The function used to transform the temperature variable prior to training so that it has support on (−∞, ∞). Defaults to NULL, as temperature is effectively already supported on this range.
pvarname A string with the name of the temperature variable in the precipitation NetCDF.
platvar A string with the name of the latitude coordinate variable in the precipitation NetCDF files. Normally this is simply ‘lat’.
plonvar A string with the name of the longitude coordinate variable in the precipitation NetCDF files. Normally this is simply ‘lon’.
pvarconvert_fcn The function used to transform the precipitation variable prior to training so that it has support on (−∞, ∞). Defaults to log.

## Structure of a trained emulator

A trained emulator can easily be stored as an R .rds object for later use. A trained emulator contains all information learned about the ESM, including components that are useful for downstream analysis, but not necessary for generating new fields of residuals:

- **griddataT**: Input training temperature data, the contents of NetCDF files in datadir and an entry for the tvarconvert_fcn. If the temperature data has been transformed with the input argument tvarconvert_fcn to trainTP(), this also includes the transformed variable data in addition to the raw variable data in the NetCDF file. The default is not to transform the temperature data (tvarconvert_fcn = NULL).
- **griddataP**: Input training precipitation data, the contents of NetCDF files in datadir and an entry for the pvarconvert_fcn. If the precipitation data has been transformed with the input argument pvarconvert_fcn to trainTP(), this also includes the transformed variable data in addition to the raw variable data in the NetCDF file. The default is to transform the precipitation data (pvarconvert_fcn = log).
- **tgav**: Global mean temperature for the grids in griddataT.
- **meanfldT**: The mean field analysis for temperature (Steps 2-3).
  - **r**: The matrix of input temperature residuals in each grid cell, in their native distribution.
  - **b** and **w**: the mean field coefficients for use in reconstructing the full temperature data.
- **meanfldP**: Analogous to meanfldT, for precipitation.
- **tfuns**: The empirical characterization of native distributions of temperature residuals for each grid cell.
  - **cdf**: A list of the empirical CDFs in each grid cell used to transform temperature residuals from the native distribution of the grid cell to *N*(0, 1).
  - **quants**: A list of the empirical quantile functions (inverse CDF) in each grid cell used to transform generated temperature residuals from *N*(0, 1) to the native distribution for temperature residuals in each grid cell.
- **pfuns**: Analogous to tfuns, for precipitation.
- **reof**: Follows the same format as the structure returned by stats::princomp(). The most important fields are:
  - **rotation**: A matrix [2 * Ngrid x NEOF] containing orthogonal basis vectors for the residuals. Each column is a basis vector. The number of basis vectors, NEOF, will be less than or equal to the number of time slices in the input, Ntime.
  - **x**: A matrix [Ntime x NEOF] containing the coordinates of the residuals in the coordinate system defined by the basis vectors. Each row is a time slice. Thus $residual\left(t\right)=\sum_{i=1}^{N}x\left[t,i\right]*rotation\left[:,i\right]$.
- **fxmag**: The magnitude of the Fourier transform of the EOF projection coefficients. This should be a matrix [Ntime x NEOF]
- **fxphase**: List of matrices [Ntime x NEOF] of phases of the Fourier transform. There should be one element in the list for each input ESM run.
- **infiles**: Names of input files used to construct the data.

## Generating new realizations

From a trained emulator, new residuals are generated (Table 1, right column, Steps 9-11) by generate.TP.resids(), which calls the fldgen functions mkcorrts(), reconst_fields(), and unnormalize.resids().

## Set RNG seed if reproducible results desired:

set.seed (11)

## Generate new residuals

residgrids <- generate.TP.resids (emulator, ngen = 5)

The function generate.TP.resids() returns an R list of new residual matrices. Each of the ngen entries in the list is a new realization: a matrix that is [Ntime x 2 * Ngrid]. The first 1:Ngrid columns of each residual matrix are the temperature residuals and columns (Ngrid + 1):(2*Ngrid) are the precipitation residuals. These generated fields can be reshaped, plotted, analyzed, saved, etc. in whatever way a user desires. With the default inputs to trainTP() of tvarconvert_fcn = NULL and pvarconvert_fcn = log, these are residuals in (temperature, log(precipitation) space.

From a trained emulator and generated new residual fields, residgrids, new full fields for temperature and precipitation are generated (Table 1, right column, Steps 12-13) by generate.TP.fullgrids().

generate.TP.fullgrids(emulator, residgrids, tgav = tgav,

         tvarunconvert_fcn = NULL,

         pvarunconvert_fcn = exp,

         reconstruction_function = pscl_apply) ->

    fullgrids

## Availability

### Quality control

Strict requirements for input files are documented. An example workflow for training an emulator and generating new residuals (using the commands outlined above) is included in the package. Using the R packages devtools and roxygen2, extensive documentation for every function created in fldgen is included. More detail of each step in Table 1 may be found in the tutorial vignettes included with the package (fldgen/vignettes/). Integrated testing (via the testthat R package and example NetCDF data included in fldgen/inst/extdata) is included and may be run with devtools::test(). The full suite of integrated tests is invoked in addition to a battery of standardized R checks with devtools::check().

### System requirements

fldgen has been tested successfully on Linux (64-bit), Windows 7 and Mac OS X.

fldgen uses multiple enormous global gridded datasets for emulator training. Therefore, a minimum memory size of 8GB is necessary, and 16GB is recommended. Memory capacity determines how fast the code is able to run, how many variables fldgen is able to analyze the joint properties for, how many scenarios fldgen may train on, and how many generated fields may be held in memory.

### Programming language

R (≥ 3.3.3).

#### Dependencies

Required dependencies include the R packages: assertthat (≥ 0.2.0), dplyr (≥ 0.7), tidyr (≥ 0.7.1), tibble (≥ 1.3.4), ggplot2 (≥ 2.2.1), scales (≥ 0.5.0), reshape2 (≥ 1.4.2), ncdf4 (≥ 1.16), rlang (≥ 0.1.2).

Optional dependencies include the R packages: testthat, gcammaptools (≥ 0.4), covr, knitr, rmarkdown.

### Software location

#### Archive

Name: zenodo

Persistent identifier: http://doi.org/10.5281/zenodo.2605386

Licence: BSD 2-Clause

Publisher: Robert Link

Version published: v2.0.0 prerelease

Date published: archive of prerelease published 25/03/2019

#### Code repository

Name: GitHub

Identifier: https://github.com/JGCRI/fldgen/tree/v2.0.0-rc.1

Licence: BSD 2-Clause

Date published: prerelease published 25/03/2019
